# Widening the reach of structural biology

**DOI:** 10.1107/S2052252516002438

**Published:** 2016-02-25

**Authors:** Edward N. Baker

**Affiliations:** a School of Biological Sciences, University of Auckland, School of Biological Sciences, Private Bag 92-019, Auckland, New Zealand

**Keywords:** structural biology, cryo-electron microscopy, free electron lasers, editorial, IUCrJ

## Abstract

An exciting future is outlined in which new approaches complementary to conventional X-ray crystallography will substantially widen the reach of structural biology. Primary among these are the recent advances in cryoelectron microscopy and the growing applications of free electron lasers.

Take a look inside any biochemistry textbook and one is presented with scores of beautiful images of biological macromolecules, ranging from the famous DNA double helix to the amazing diversity of proteins. Look behind these images into the atomic detail that is archived in the Protein Data Bank and one can start to discover how the processes of biology take place. These atomic level structures – more than 100 000 at the present time – provide a rich resource for understanding biology and a knowledge base for applications in biotechnology and medicine.

Historically, some 90% of these structures have come from X-ray crystallography, which has in the process contributed to more than a dozen Nobel prizes. These are achievements in which the crystallographic community can take great pride. At the same time, science does not rest, and articles in this journal and others have given us glimpses into an even more exciting future, in which complementary approaches will substantially widen the reach of structural biology. Primary among these are the new advances in cryo-electron microscopy (cryoEM) (reviewed by Subramaniam *et al.*, 2016 ▸) and the growing applications of free electron lasers (FELs) (Schlichting, 2015 ▸). Both approaches have a natural home in this journal.

CryoEM studies have had a long gestation and for some years the method appeared to be limited to moderate resolutions of 6–8 Å, too low to give atomic detail, except in cases where crystal structures could be docked into the EM envelope. Spectacular advances have, however, come in the past two years with the development of new detectors (direct electron detectors) that have literally revolutionized the field (Subramaniam *et al.*, 2016 ▸). Many complex biological systems that could not be accessed before – generally because crystals suitable for X-ray crystallography could not be prepared – are now within reach of high-resolution structural analysis: large multi-component biological machines and complexes, membrane proteins and the like. Moreover, single-particle analysis of cryoEM images also enables flexible systems to be seen in multiple conformations, allowing unprecedented views of dynamic changes that are critical to biological function. Time will tell what the ultimate limits of cryoEM may be, but already resolutions as high as 2.2 Å have been attained (Bartesaghi *et al.*, 2015 ▸).

In contrast, FELs are of much more recent impact on structural biology, but have already made remarkable advances (Schlichting, 2015 ▸). Until some 15 years ago applications to biological molecules were little more than a gleam in the eye of a few visionaries (Neutze *et al.*, 2000 ▸). The development of the first FEL sources in the past five years or so has, however, enabled some of these dreams to be realised. As one example, articles in **IUCrJ** have tracked the growth in applications of serial femtosecond crystallography (SFX) in which whole data sets can be derived from thousands of diffraction shots, each taken from a protein crystal before it is blown apart. This approach has the advantage that the data are recorded before any radiation damage occurs, a feature that can be crucial for understanding molecular function. The use of FELs for structural biology is still in its infancy, but with exciting results for at least one membrane protein (Nogly *et al.*, 2015 ▸) and a promising recent example of diffraction from crystals formed inside cells (Jakobi *et al.*, 2016 ▸), this is another exciting option for the future.

It seems to me, however, that these advances do not in any way diminish the power and relevance of conventional X-ray crystallography in the fields of biology and medicine. Conventional protein crystallography remains a bedrock of modern drug development, providing the atomic detail necessary for discovery and optimization of new drug candidates. The high resolution that is potentially attainable from even the smallest crystals – especially with modern synchrotron beamlines (Helliwell & Mitchell, 2015 ▸) – is essential for translating structural observations into chemical interpretations. Moreover, new developments in this field, too, continue to enhance it: use of robotics has revolutionized crystallization, automation in structure determination is well advanced, new phasing methods continue to emerge and the rigorous validation tools developed for crystal structures provide a model for other approaches to structural analysis.

A feature of structural biology today is that it is a goal-oriented discipline. With many options to choose from, researchers can now choose whichever approaches will best enable them to address the questions they want to answer. It has become common, for example, for researchers to use crystallography to determine the structure of a protein and NMR to study dynamic aspects relevant to function. Solution scattering techniques such as SAXS can be used to visualize the conformations taken up by multi-domain proteins or multi-protein complexes, and are particularly useful when the structures of components are already known from crystallography. CryoEM will provide the ideal approach for some purposes, FELs for others. And there will be cross-fertilization; already, serial crystallography techniques developed for FELs are being migrated to synchrotron beamlines.

Our goal for this journal, now in its third year of publication, is that it should cover the full richness of structural biology across the whole range of approaches discussed here, as well as the exciting new findings that are emerging across many sectors of biology and medicine. A selection of papers in this field can be found at http://journals.iucr.org/m/services/articles_biol_med.html.
